# Preparation and Characterization of Poly(ether-block-amide)/Polyethylene Glycol Composite Films with Temperature-Dependent Permeation

**DOI:** 10.3390/polym10020225

**Published:** 2018-02-24

**Authors:** Sarinthip Thanakkasaranee, Dowan Kim, Jongchul Seo

**Affiliations:** Department of Packaging, Yonsei University, 1 Yonseidae-gil, Wonju-si, Gangwon-do 26493, Korea; s.thanakkasaranee@gmail.com (S.T.); dodo2863@naver.com (D.K.)

**Keywords:** phase change materials (PCMs), poly(ether-block-amide) (PEBAX), polyethylene glycol (PEG), controllable permeability, permeation jump

## Abstract

A series of poly(ether-block-amide) (PEBAX)/polyethylene glycol (PEG) composite films (PBXPG) were prepared by solution casting technique. This study demonstrates how the incorporation of different molecular weight PEG into PEBAX can improve the as-prepared composite film performance in gas permeability as a function of temperature. Additionally, we investigated the effect of PEG with different molecular weights on gas transport properties, morphologies, thermal properties, and water sorption. The thermal stability of the composite films increased with increasing molecular weight of PEG, whereas the water sorption and total surface energy decreased. As the temperature increased from 10 to 80 °C, the low (L)-PBXPG and medium (M)-PBXPG films showed a trend similar to the pure PEBAX film. However, the high (H)-PBXPG film with relatively high molecular weight exhibited a distinct permeation jump in the phase change region of H-PEG, which is related to the temperature dependent changes in the morphology structure such as crystallinity and the chemical affinity between the polymer film and gas molecule. Based on these results, it can be expected that H-PBXPG composite films can be used as self-ventilating materials in microwave cooking.

## 1. Introduction

The microwave market is expected to witness a remarkable growth fueled by consumer demands due to the easy preparation and portability of on-the-go eating. The important driving factors for this growth are not only improvements in packaging materials and design that permit food to be heated in the package itself and served instantly, but also the innovative technologies that overcome the limitations of microwave ovens like browning and crisping [1].

The safety issues of microwave ovens involve setting the correct combination of time and temperature when cooking a pre-packaged food product to prevent it from burning, and also using a proper package that can heat at high temperature and release steam [2]. However, most packaged foods are not automatically self-venting during microwave cooking, which can cause the package to explode due to an excessive internal pressure buildup. Therefore, the user has to partially lift the plastic sheet from the tray or puncture the plastic covering to provide an outlet for the steam that is generated by the heated food items. Nonetheless, such a problem can be solved by improving the design and quality of the packaging materials or using innovative technologies to obtain microwaveable packaging with self-ventilation during the cooking process. This idea would not only solve this issue, but also meet user demand by increasing convenience and safety, since users would not be required to manually prepare or enable the venting mechanisms thus eliminating the chances of cuts and burns [3].

Self-venting materials have been known to deal with issues like the ones described below: A weak heat seal that fractures from excess steam pressure during microwave steam generation; incorporation of shrink-film-covered vent valves that melt or otherwise fail due to steam pressure; or laser-scored or perforated film which fails from the internal steam-pressure and releases the steam in the microwave oven [4]. However, multiple processes are required to produce such microwavable packages, leading to relatively high production costs. Innovative technologies like the controllable permeability technology may be an alternative to solve these issues.

Recently, controllable permeability technologies like developing polymers with temperature-dependent permeability are applied in many fields including membranes, drug delivery, and packaging (i.e., agricultural products, and medical devices) [5]. Therefore, studying the temperature dependence on gas or water vapor permeability of the polymer materials is crucial to food packaging in different or uncontrollable environmental conditions [6,7,8]. Generally, polymer films with temperature-dependent permeability are non-porous structures without pinholes, allowing water vapor or gas to pass through due to the morphological and chemical structure of the polymer material itself. Many polymers with temperature-dependent permeability have been studied, including thermoplastic polyurethanes, poly(*N*-isopropylacrylamide), and poly(ether-block-amide) (PEBAX). The application of these polymer films (non-porous structures) in food packaging can minimize the problems of quality loss and food deterioration usually originating from the porous structure of some polymer composite films under storage, distribution, and sales environments. The polymer films with temperature-dependent permeability can be positively modulated to achieve optimum qualities by controlling the gas or vapor permeability depending on the temperature changes of the environmental conditions [8,9]. 

PEBAX is a well-known block copolymer consisting of a polyamide block as a hard segment and a polyether block as a soft one. The crystalline amide block in PEBAX functions as an impermeable phase, whereas the ether block acts as the permeable phase due to its high chain mobility [10]. PEBAX has an excellent gas separation property for polar/nonpolar gas mixtures [11]. PEBAX also allows water vapor molecules to diffuse, while simultaneously blocks the transport of liquid water [12]. Thus, it can be used as a functional material in sportswear, food packaging, and medical packaging applications for gas sterilization [13].

The introduction of phase change materials (PCMs) into the polymer matrix is one feasible approach to control the gas or water vapor permeability responding to the desired temperature changes under the external atmosphere of packaging. PCMs are temperature-responsive substances that absorb and release large amounts of latent heat energy during temperature-driven phase changes [14]. Among various PCMs, polyethylene glycol (PEG) has attracted great interest due to its good characteristics such as various phase change temperatures depending on the molecular weight, congruent melting, non-toxicity, small or no volume changes during solid–liquid phase changes, and high thermal and chemical stability after long-term service. In addition, PEG can be directly incorporated into porous materials [15], it has a melting temperature around 3.2–68.7 °C, and a very high phase change enthalpy depending on its molecular weight [16]. Polyethylene glycol can possibly allow the development of composite films with optimal gas or water vapor permeability at different targeted temperature ranges. The composite films consisting of two different phases of polymer and PCM, and the transformation of this latter into a polymer matrix occurring near the PCM phase temperature can significantly affect the morphology of the entire polymer/PCM composite film, and effectively create passing channels for various penetrants in the polymer matrix.

According to the above issues, the development of composite films with temperature-dependent permeability by introducing PCMs is a possible candidate for microwaveable packaging materials with controllable permeability properties that can be safe and easy to use. The unique property of the polymer/PCM composite films may create enough passing channels for the excessive internal gas or vapor buildup in packages during microwave cooking. In this work, we proposed to develop a polymer/PCM composite film packaging material with a temperature-dependent gas permeability, which could be used as a self-ventilating microwaveable material. This could be amenable to food packaging applications by preventing packaging damages and explosions during the cooking process. A series of (PEBAX)/(PEG) composite films (PBXPG) were prepared by the solution casting technique and their gas permeability, morphologies, thermal properties, and water sorption properties were investigated as a function of PEG with different molecular weights.

## 2. Materials and Methods

### 2.1. Materials

PEBAX MH 1657, consisting of 60 wt % poly(ethylene oxide) (PEO) and 40 wt % polyamide 6 (PA-6) was purchased from Arkema Co. Ltd. (Paris, France). Three grades of PEG, PEG950–1050 (average *M*n 950–1050), PEG3350 (average *M*n 3,350), and PEG35000 (average *M*n 35,000), were purchased from Sigma-Aldrich Korea Ltd. (Yongin, Korea). Ethyl alcohol (94 wt %) was purchased from SK Chemicals Co., Ltd. (Gyeonggi, Korea). All materials in this study were used as received.

### 2.2. Preparation of PEBAX, L-PBXPE, M-PBXPE, and H-PBXPE Composite Films

First, 10 g of PEBAX was dissolved at a 10 wt % concentration in a mixture of ethanol/water (70/30 wt %) under reflux at 80 °C and stirring for 2 h. An equal amount of PEG was added to the PEBAX solution and stirred for 1 h. The obtained PEBAX and PEG mixture was casted onto a glass substrate using a bar-type automatic film coating apparatus (KIPAE E&T Co. Ltd., Hwasung, Korea). The films were dried in a vacuum oven at 30 °C for 24 h and then at 70 °C for 12 h. All films were maintained at a 100 ± 3 μm thickness to aid in evaluating their physical properties. Depending on the molecular weight of PEG, the composite films were designated as follows; L-PBXPG for PEG950–1050, M-PBXPG for PEG3350, and H-PBXPG for PEG35000, respectively.

### 2.3. Characterization

#### 2.3.1. Fourier Transform Infrared (FT-IR)

To characterize the chemical structure of pure PEBAX and composite films, FT-IR spectra were recorded on a Spectrum 65 FT-IR spectrometer (PerkinElmer Co., Ltd., Waltham, MA, USA) from 4000 to 400 cm^−1^ in an attenuated total reflection (ATR) mode with diamond/ZnSe (1 reflection) crystal.

#### 2.3.2. Scanning Electron Microscopy (SEM)

To investigate the surface morphology of the pure PEBAX and composite films, we obtained SEM images for the top and fractured surfaces using a Quanta FEG250 scanning electron microscope (FEI Co., Ltd., Hillsboro, OR, USA) at acceleration voltage of 30 kV and working distance of 10 mm. The fractured surfaces of the films were analyzed using specimens obtained after a tensile test. Prior to examination, all samples were coated with a thin layer of Pt/Pd.

#### 2.3.3. Thermogravimetry (TGA) and Differential Scanning Calorimetry (DSC)

We measured the thermal properties for different PEGs, pure PEBAX and the composite films using a Q10 differential scanning calorimeter (TA Instrument Co. Ltd., New Castle, DE, USA). Samples of approximately 5 mg were heated from −80 to 220 °C at a heating rate of 10 °C/min in a nitrogen atmosphere (20 mL·min^−1^). We also tested the thermal stability of the composite films using a TGA 4000 thermogravimetric analyzer (Perkin Elmer Co. Ltd., Waltham, MA, USA). The measurements were performed in a temperature range of 30 to 800 °C at a heating rate of 10 °C/min in a nitrogen atmosphere (20 mL·min^−1^). The mass of the samples was about 10 mg.

#### 2.3.4. Universal Testing Machine (UTM)

We measured the mechanical properties of the composite films using a universal testing machine QM 100T (Qmesys Co. Ltd., Kwangmyeong, Korea) and compared them with the pure PEBAX film. The measurements were performed at a test speed of 500 mm/min for specimens that were 8 cm × 1 cm. We analyzed the resulting profiles using a MC tester version 12.6 (Qmesys Co., Ltd., Kwangmyeong, Korea).

#### 2.3.5. Dynamic Vapor Sorption (DVS)

We performed water sorption of the composite films using a DVS Intrinsic (Surface Measurement Systems, London, U.K.) equipped with SMS UltraBalance^TM^ with a mass resolution of ± 0.1 μg. Typically, we placed 5 mg samples into the sample pan and dried them under a flow of dry nitrogen at 25 °C and 0% relative humidity (RH) for 5 h. After that, we tested the samples at 25 °C and 95 % RH for 24 h. We obtained the water uptake by measuring the change in mass with respect to the dry mass.

#### 2.3.6. Contact Angle and Surface Free Energies

We measured the contact angles and surface free energies of the composite films using a Phoenix 300 contact angle goniometer (SEO Co. Ltd., Suwon, Korea). We carried out the measurements using deionized water and methylene iodide on samples at 25 °C, and we observed the changes via an eyepiece by applying the θ/2 method. We maintained the volume of the sessile drop as 5 μL in all cases by using a micro syringe. We averaged the contact angles for five drops and calculated the surface free energies applying the harmonic-mean equation [17] using the contact angles for water and methylene iodide on the composite films.

#### 2.3.7 Oxygen Transmission Rate (OTR)

We measured the OTR of the composite films using a BT-1 gas transmission rate tester (Toyo Seiki Seisaku-sho, Ltd., Tokyo, Japan) at different temperatures ranging from 10 to 80 °C and a 0% RH in the chamber.

## 3. Results and Discussion

### 3.1. Preparation of Composite Films

**Figure 1:**
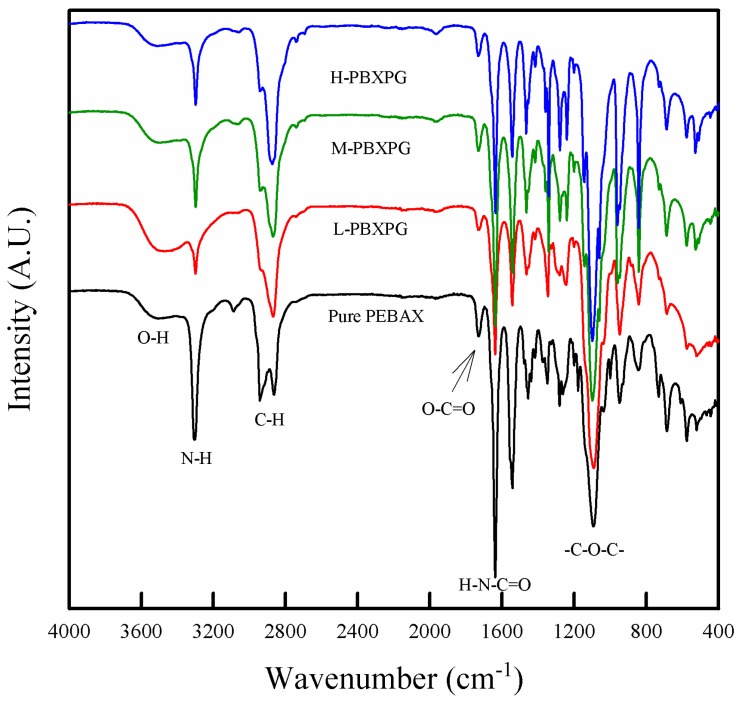
Fourier Transform Infrared (FT-IR) spectra of the pure poly(ether-block-amide) (PEBAX) and composite films.

We studied the incorporation of different molecular weight PEG into the PEBAX matrix via FT-IR, as illustrated in Figure 1. The pure PEBAX film exhibited several strong bands: 1093 cm^−1^ corresponding to the –C–O–C– group in the PEO segment, 1637, 1730, and 3306 cm^−1^ indicating the –HNCO–, O–C=O, and –NH– group in the PA-6 segment, 2866 cm^−1^ representing –CH_2_– in the soft and hard segment, and 3500 cm^−1^ for the terminal –OH group in PEBAX, respectively [18]. Although the composite films showed almost the same bands as pure PEBAX, there were some shifts in the characteristic peaks depending on the PEG introduction. The –NH– peak at 3306 cm^−1^ for pure PEBAX apparently shifted to lower wavenumbers: 3299 cm^−1^ for L-PBXPG, 3298 cm^−1^ for M-PBXPG, and 3298 cm^−1^ for the H-PBXPG composite films, whereas the –C–O–C– peak at 1093 cm^−1^ for pure PEBAX evidently moved to higher wavenumbers: 1095 cm^−1^ for L-PBXPG, 1099 cm^−1^ for M-PBXPG, and 1098 cm^−1^ for the H-PBXPG composite films, respectively. These changes happen due to the disruption of the existing interchain hydrogen bonding in PEBAX [19] and the formation of new hydrogen bonds between PEG and PEBAX [20]. PEG provides a relatively high population of –OH groups, which could interact with the –OH, –NH–, and –HNCO– groups and reduce the attractive intermolecular forces (hydrogen bonding) in PEBAX. It is expected that the composite films show a relatively good dispersion of PEG in the PEBAX matrix. Besides, a decrease in the intensity of the –OH peak with increasing molecular weight of the PEG in the composite films is likely to cause a decrease in the hydrophilicity of the composite films.

### 3.2. Morphology

**Figure 2:**
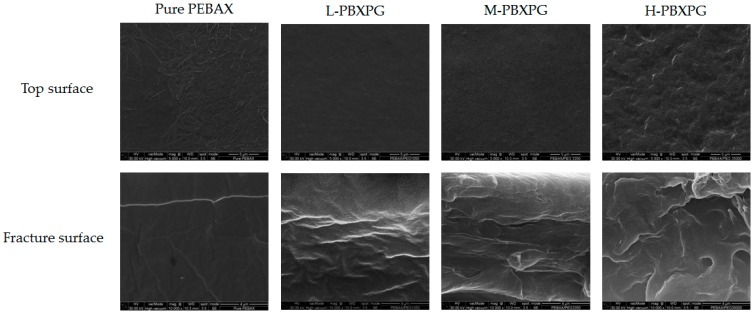
Scanning Electron Microscopy (SEM) images of the pure PEBAX and composite films.

SEM analysis is frequently performed to determine the dispersion and miscibility in the composites, which generally affect their thermal stability, mechanical properties, and permeation properties [21,22,23]. As shown in Figure 2, the pure PEBAX film had a relatively rough appearance and a long ribbon form resembling a nanofibril on the top surface, which might be related to the two separated microphases of PEBAX. The nanofibril morphology might originate from the oriented polymer lamella and crystalline nature of PA-6 [24,25,26]. The L-PBXPG and M-PBXPG composite films showed relatively smooth surfaces, whereas the H-PBXPG composite film exhibited a relatively rough surface. These occur due to the low molecular weight PEGs with high chain mobility, which act as plasticizers and increase the chain mobility of PEBAX. This results in a smooth surface in the composite films with low molecular weight PEGs [27]. High molecular weight PEGs with low chain mobility seem to inhibit the rearrangement of the PEBAX chain, resulting in a relatively rough surface. In the tensile fracture surface SEM images, the pure PEBAX film showed a uniform and regular failure surface with an embossed line, which could be attributed to the plastic deformation of the pure PEBAX [28]. Morphology changes are more distinguishable for the composite films with higher molecular weight of PEG. The H-PBXPG film exhibited the most irregular and rough failure surface of all those composite films. This might be related to the chemical interaction and difference in flowability between PEG and PEBAX. The composite films show good interaction between PEG and PEBAX, as described in FTIR. However, the high molecular weight PEG has difficulty flowing in the PEBAX matrix, resulting in a very rough surface and noticeable plastic deformation under tensile stress. This result can be expected to cause a decrease in mechanical properties. 

### 3.3. Thermal Properties

**Figure 3:**
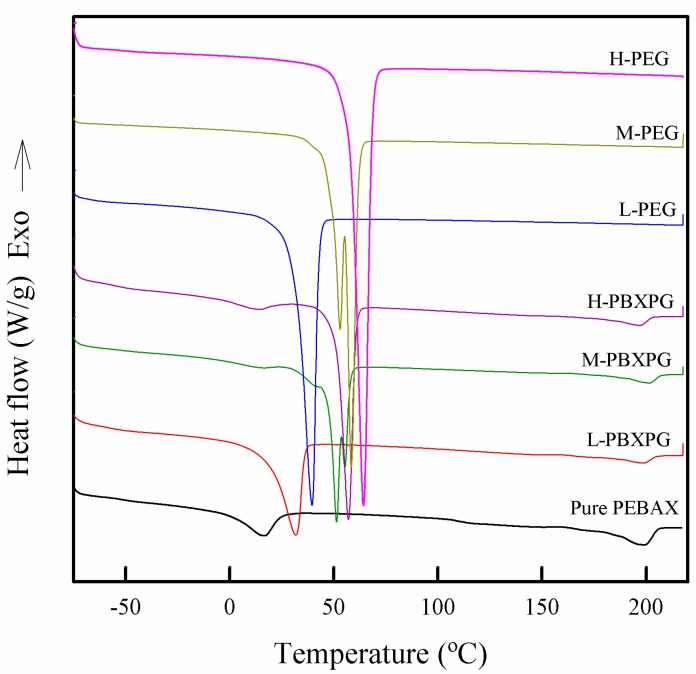
Differential Scanning Calorimetry (DSC) curves of the pure PEBAX and composite films.

The crystalline phase of a polymer composite plays a significant role in determining its physical properties [29], and the degree of crystallinity can be generally estimated using the heat of fusion in a DSC thermogram [30]. Figure 3 shows DSC curves of prepared composites and the values associated with the thermal phenomena are reported in Table 1. Regardless of the molecular weight, all PEGs showed one melting temperature (*T*_m1_): 40 °C for L-PEG, 58 °C for M-PEG, and 64 °C for H-PEG, respectively. However, the pure PEBAX film displayed two melting temperatures at 16 and 199 °C corresponding to the fusion of the crystalline fraction of the blocks PEO and polyamide (PA), respectively [24]. All the composite films also showed two melting temperatures, similar to the pure PEBAX film. The *T*_m1_ and heat of fusion (∆*H*_m1_) of the L-PBXPG, M-PBXPG, and H-PBXPG composite films increased from 16 to 32, 51, and 57 °C, and 33 to 68, 87, and 95 J/g, respectively. Apparently, the lower *T*_m1_ and ∆*H*_m1_ for the composite films increased with increasing molecular weight of PEG. This result indicates that there is an increased tendency for higher molecular weight PEG to form the crystalline phase due to the lower segmental mobility and more convenient geometrical alignments [31]. The higher melting temperature (*T*_m2_) of the pure PEBAX and composite films occurred around 200 °C due to the melting of the PA domains [32]. Comparing *T*_m1_ and ∆*H*_m1,_ with *T*_m2_ and ∆*H*_m2_, they showed different dependencies on the molecular weight of PEGs. The incorporation of PEGs into the PEBAX films did not influence *T*_m2_, but affected the ∆*H*_m2_ of the PA domains by reducing it from 35 J/g for pure PEBAX to 5 J/g for L-PBXPG, 6 J/g for M-PBXPG, and 9 J/g for the H-PBXPG composite films, respectively. The reduction of ∆*H*_m2_ indicates that the crystallinity of the composite films is highly deteriorated, and that is related to the disruption of the interchain hydrogen bonding in the PA domain itself [19], as explained by the FT-IR results.

**Figure 4:**
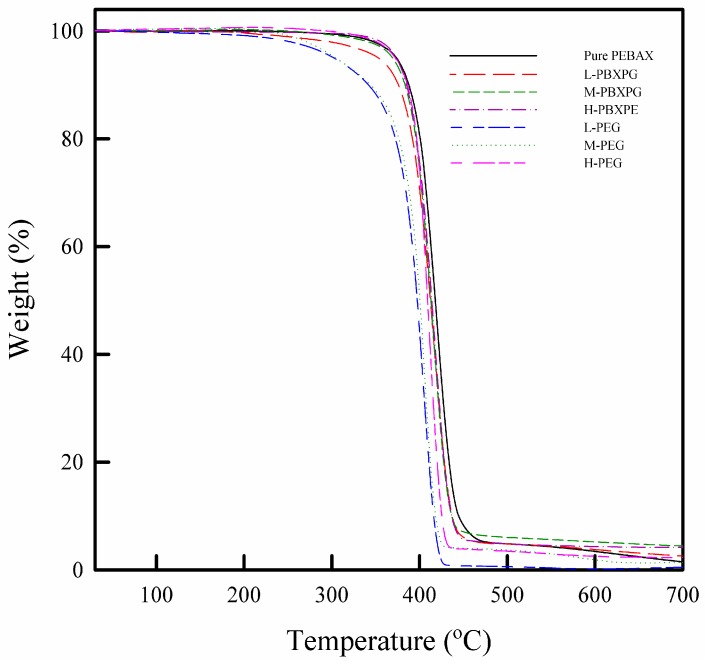
Thermogravimetry (TGA) curves of the pure PEBAX and composite films.

Figure 4 shows TGA curves of the pure PEBAX and composite films. All the PEGs and PEBAX showed one-step decomposition. The decomposition temperature is the temperature at which the substance chemically decomposes [33]. The decomposition temperature at 1%, 3%, and 5% mass loss in PEG, pure PEBAX, and composite films are reported in Table 1. The decomposition temperature increased with increasing molecular weight of PEG. However, the composite films showed a strong dependence of molecular weight on the thermal stability. The thermal degradation of PEG and PEBAX is related to the random chain scission mechanism of the main chain [34,35]. The composite films showed one-step decomposition, similar to the pure PEBAX, indicating that the presence of the PEG does not significantly influence the thermal degradation pattern in the composite films. However, the composite films showed higher thermal stability for higher molecular weight PEG. This result is related to the thermal stability of PEGs depending on their molecular weight. High molecular weight PEGs with relatively high thermal stability delay the initial thermal degradation in the thermally unstable PEO blocks.

### 3.4. Mechanical Properties

**Figure 5:**
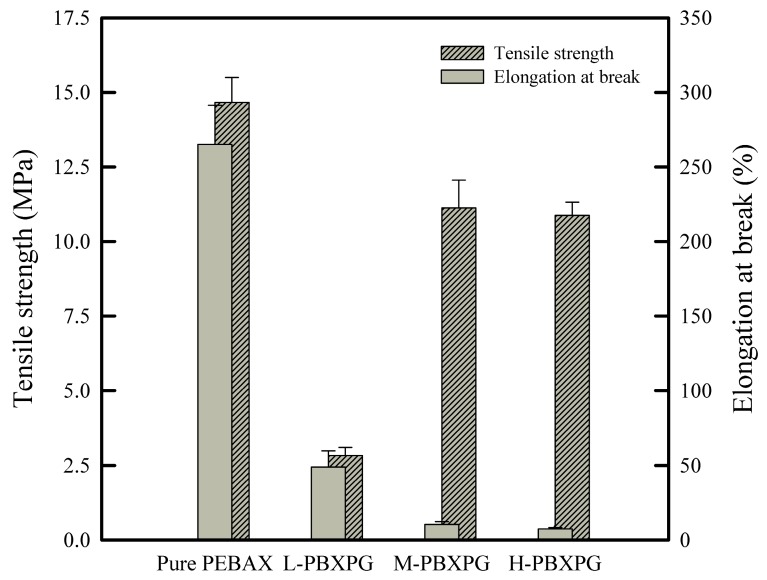
Tensile strength and elongation at break of the pure PEBAX and composite films.

PEBAX has flexible PEO and rigid PA-6 segments. The crystalline PA-6 domains provide mechanical strength and act as intermediate spacers between the PEO domains hindering their crystallization and offering greater chain mobility by the ether linkage [32]. As shown in Figure 5, the introduction of PEGs into PEBAX induced an increase in both tensile strength and elongation at break in the composite films. As described by FT-IR, PEGs have good interaction with PEBAX by forming hydrogen bonds with the PEBAX molecule and disrupting the interchain hydrogen bonding between the PA-6 domains, resulting in a reduction of crystallinity in PA-6. As a consequence the composite films lose their mechanical strength and elasticity [36,37]. However, the composite films showed a strong dependence of the mechanical properties on molecular weight. In general, amorphous regions provide certain elasticity, whereas the mechanical strength of the materials depends on the degree of crystallinity. Higher crystallinity results in a harder material with more brittle properties. As expected, the composite films showed lower elongation at break, but higher tensile strength with higher molecular weight PEG. This result is related to the crystallinity of PEGs depending on their molecular weight. Higher molecular weight PEGs with relatively higher crystallinity effectively enhanced the crystallinity in the PEO segment of PEBAX, as reflected by the ∆*H*_m1_, and shown in the DSC results. The increased crystallinity of the composite films caused more brittleness, which brings about an increase in mechanical strength and a decrease in elongation at break in the composite films. 

### 3.5. Water Sorption and Surface Free Energy

**Figure 6:**
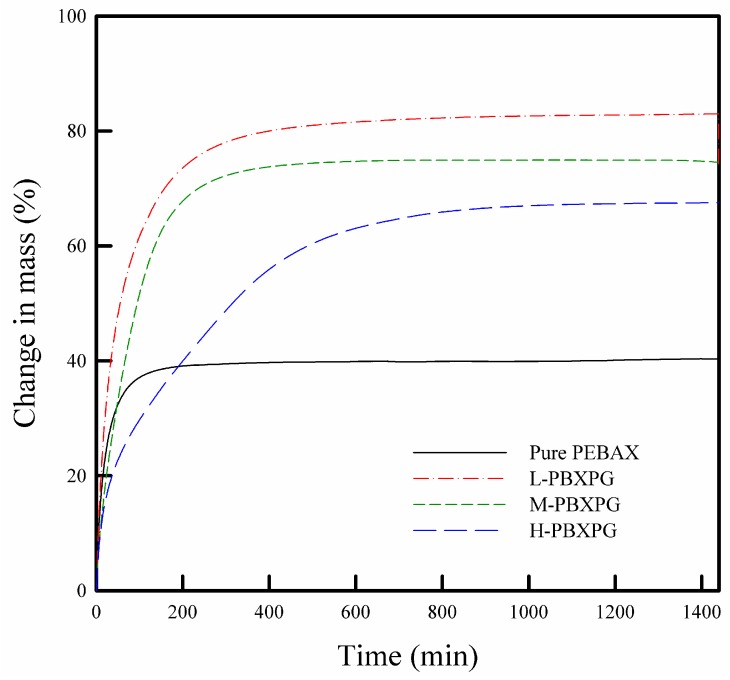
Water sorption isotherms of the pure PEBAX and composite films measured at 95 % relative humidity (RH) and 25 °C.

As shown in Figure 6, the pure PEBAX film showed lower water uptake than the composite films. With increasing molecular weight of PEG, the water uptake and the time to get saturated decreased, indicating that the water sorption behavior strongly depends on the molecular weight of PEG. Usually, the water sorption behaviors of polymer films are correlated with the chemical and/or morphological structure [38,39]. The water uptake relies on the chemical affinity to water (hydrophilicity). As expected, the composite films with higher molecular weight PEG showed lower water uptake because of a lower hydroxyl group’s population, as explained in the FT-IR spectra. Besides, the rate of water uptake depends on the morphological structure, which relates to crystallinity and/or free volume. The composite films with higher molecular weight PEG showed a lower diffusion rate, i.e., longer time to get saturated due to higher crystallinity. H-PBXPG has the highest crystallinity as reflected by the heat of fusion of 95 J/g for the PEO segment and 9 J/g for the PA-6 segment, as depicted in Table 1. 

**Figure 7:**
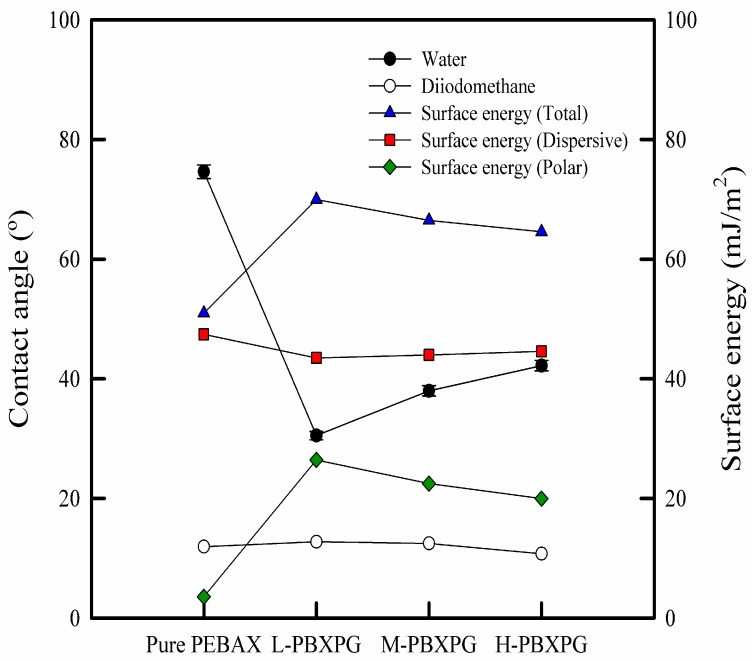
Contact angle and surface energy of the pure PEBAX and composite films.

As shown in Figure 7, the water contact angle of the composite films showed strong dependence on the introduction of PEG. However, the incorporation of PEG into the PEBAX matrix did not affects the diiodomethane contact angle. The contact angle is mostly affected by the chemical structure (polar and nonpolar groups) and morphology (surface roughness) of the polymer surface [40]. PEBAX and PEG are hydrophilic polymers, and the polar groups easily interact with water molecules, hence, that obviously influences the water contact angle. With increasing molecular weight of PEG, the water contact angle of the composite films increased, indicating that the composite films become less hydrophilic. The total surface energies of the composite films were determined by the Owens–Wendt method (geometric mean combining rule), which utilizes the contact angle measurement of deionized water and diiodomethane [41]. In general, the total surface energy decreases when hydrophobicity increases [42]. In this work, the total surface energy of the composite films decreased with increasing molecular weight of PEG, showing that the hydrophilicity of the composite films also decreased. Higher molecular weight of PEG has less polar (–OH) groups, leading to a lower total surface energy. The results of contact angle and water sorption confirm that the hydrophilicity of composite films decreases with increasing molecular weight of PEG. 

### 3.6. Oxygen Permeability

**Figure 8:**
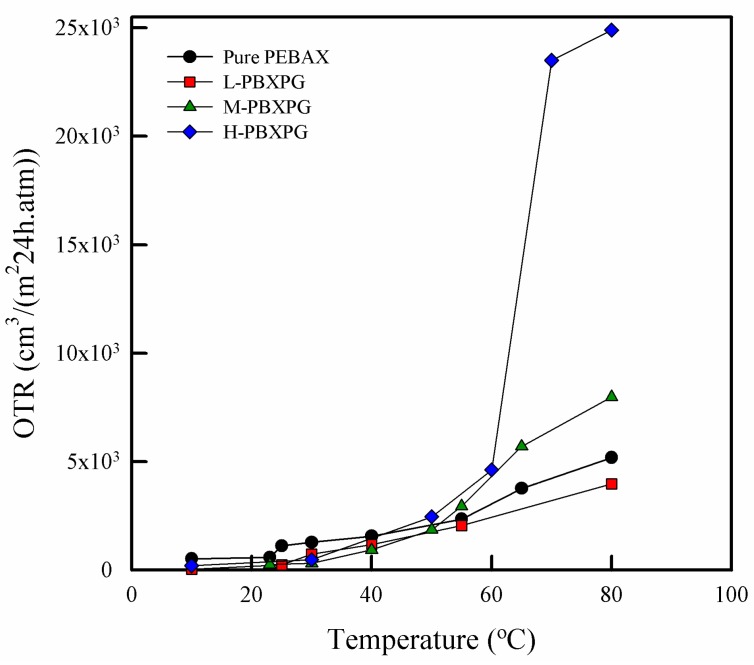
Oxygen transmission rate of the pure PEBAX and composite films.

As shown in Figure 8, the OTR of the pure PEBAX film was 521 cm^3^/(m^2^·24h·atm) at 10 °C, whereas the composite films showed 34.4 cm^3^/(m^2^·24h·atm) for L-PBXPG, 254 cm^3^/(m^2^·24h·atm) for M-PBXPG, and 201 cm^3^/(m^2^·24h·atm) for H-PBXPG, respectively. Obviously, the composite films showed lower values than the pure PEBAX film. With increasing temperature from 10 to 80 °C, the pure PEBAX film showed a linear increase from 521 to 5190 cm^3^/(m^2^·24h·atm). The L-PBXPG and M-PBXPG films also showed a linear increase in OTR from 39.4 to 3970 cm^3^/(m^2^·24h·atm), and from 254 to 7980 cm^3^/(m^2^·24h·atm), respectively. However, the H-PBXPG film showed an abnormally large increase from 201 to 24,900 cm^3^/(m^2^·24h·atm). The OTR of H-PBXPG film slowly increased with increasing temperature from 10 to 60 °C and distinctly jumped when the temperature reached approximately 65 °C. This demonstrates that the oxygen permeability of the composite films may depend on the molecular weight of PEG. The permeation of gases through polymer films can be usually explained in terms of a ‘‘solution-diffusion’’ mechanism [43] which consists of the following steps [44]: (1) solution (absorption) of small molecules into the film at the side of the higher potential (pressure, concentration, etc.); (2) molecular diffusion of the molecules in and through the film; and (3) release (desorption) of the diffused molecules from the solution at the opposite side into the liquid or gas phase at lower potential. The gas permeability of the polymer relies on many factors like the degree of crystallinity, molecular weight, and chemical affinity [45]. As described in the introduction section, the PEO segment acts as a permeable phase due to its high chain mobility and PEG is a very ordered structure, which is easy to crystallize. This will create a semicrystalline soft phase and reduce the mass transport properties in the film or membrane [24]. As expected, the composite films showed a lower OTR at temperatures below *T*_m1_ than the pure PEBAX film. The composite films have higher crystallinity (lower free volume) than the pure PEBAX film, therefore gas molecules cannot easily diffuse through them. The molecular weight of the polymer is related to the number of chain ends, which also influences the gas permeability. The chain ends represent discontinuities and may form sites for the gas molecules to be absorbed, diffused, and desorbed through the polymer film. As the molecular weight of the polymer increases, the number of chain ends also decreases, resulting in a decrease in gas permeability. Generally, gas permeability decreases when the crystallinity and molecular weight of the polymer increase. However, our results show a contrary trend: the H-PBXPG film with a higher molecular weight of PEG and crystallinity has a higher OTR than the M-PBXPG and L-PBXPG films. This phenomenon is related to the chemical affinity (functional group) between the polymer films and gas molecules. The hydroxyl terminal groups (polar groups) in PEG have a weak affinity to oxygen molecules (non-polar gas), resulting in a lower solubility and permeability of oxygen. As the molecular weight of PEG increases, the number of hydroxyl terminal groups of the films decreases too, leading to a higher solubility of oxygen. Therefore, the H-PBXPG film with higher molecular weight PEG showed a higher OTR than the M-PBXPG and L-PBXPG films.

When increasing the temperature above *T*_m1_ up to 80 °C, the crystalline structure of the PEO segment starts to convert to an amorphous structure. Also, the intramolecular interaction collapses during the phase change process of PEG in the vicinity of the phase transition temperature. These occurrences contribute to an increase in the chain mobility and free volume. As a result, the composite films seem to open the diffusion path for oxygen molecules [46,47,48], which leads to a permeation jump. The L-PBXPG and M-PBXPG films showed a steady increase in OTR similar to the pure PEBAX film. However, the H-PBXPG film apparently exhibited a distinct permeation jump in the vicinity of the phase change of H-PEG. The H-PBXPG film contains H-PEG (*M*n ~ 35,000), which is approximately 10 to 30 times higher than M-PEG (*M*n ~ 3350) and L-PEG (~950–1050), respectively. This dissimilarity can affect the chemical affinity for oxygen molecules, which influences the solubility of oxygen, as mentioned above. Based on these factors, the H-PBXPG film with high oxygen solubility exhibited a big permeation jump in the vicinity of the phase transition temperature of H-PEG. The unique properties like a big permeation jump and gas controllable permeability with temperature dependence can be potentially as self-ventilation for microwavable food products. 

In this study, the composite film showed a low OTR value at the low temperature. The low permeation properties of the composite films can be maintained the quality and shelf life of prepared food under storage, distribution, and sale environment condition. Some food products such as seafood generate internal steam around 62 °C during microwave heating [49]. The H-PBXPG film showed a big permeation jump at the vicinity of 65 °C. It is highly anticipated that this composite film can be used as a potential packaging material with self-ventilation to release internal steam during microwave cooking. 

## 4. Conclusions

In this study, we prepared a series of PBXPG composite films with different molecular weight of PEG for microwave packaging applications. Apparently, incorporation of PEG into PEBAX film lead to changes in morphology and chemical structure in the composite film, which affected thermal stability, mechanical properties, water sorption, surface properties, as well as permeability, depending on the molecular weight of PEG. As the molecular weight of PEG increased, thermal stability and mechanical strength increased. Further, the water vapor uptake and rate of water vapor diffusion in the composite films decreased, whereas the gas permeability increased. This demonstrates that the gas permeability and water sorption properties of composite films can be controlled by introducing different molecular weight of PEG. Among composite films, the H-PBXPG composite film with a relatively high molecular weight apparently showed a big permeation jump near the phase transition temperature of H-PEG (~65 °C). This temperature is very near the internal steam temperature (~62 °C) in some food products. Such unique properties like a big permeation jump and gas controllable permeability with temperature dependence achieved by incorporating H-PEG into the PEBAX film lead us to conclude that these films can be potentially used as packaging materials with self-ventilation by releasing internal steam for microwavable food products. 

## Figures and Tables

**Table 1:** Thermal properties of the pure PEBAX and composite films.

Code		DSC				TGA			
	*M*n (mol wt) ^a^	*T*_m1_ (°C) ^b^	∆*H*_m1_ (J/g) ^d^	*T*_m2_ (°C) ^c^	∆*H*_m2_ (J/g) ^e^	*T*_d1%_ (°C) ^f^	*T*_d3%_ (°C) ^f^	*T*_d10%_ (°C) ^f^	Residual content (%) ^g^
L-PEG	950–1050	40	135	-	-	210	274	342	0
M-PEG	3350	58	141	-	-	254	284	342	1
H-PEG	35000	64	180	-	-	333	361	386	2
Pure PEBAX	-	16	33	199	35	323	360	388	2
L-PBXPG	-	32	68	199	5	245	324	376	3
M-PBXPG	-	51	87	202	6	312	356	383	4
H-PBXPG	-	57	95	197	9	337	364	385	4

^a^ Number average molecular weight of L-PEG, M-PEG, and H-PEG. ^b,c^ Melting temperature of PEG, pure PEBAX, and composite films, respectively. ^d,e^ Melting enthalpy of PEG, pure PEBAX, and composite films, respectively. ^f^ 1 %, 3 %, and 5 % decomposition temperature of PEG, pure PEBAX, and composite films. ^g^ Residues of remained at 700 °C.
